# Effects of *Momordica cochinchinensis* Aril Extract on Sterilized Low-Fat Milk, Antioxidant and Antiproliferative Activities

**DOI:** 10.1155/2022/7934454

**Published:** 2022-02-23

**Authors:** Thitiporn Anunthawan, Santi Phosri, Jirayus Woraratphoka, Rasapirose Somwatcharajit, Panee Terdsaksri, Prangruethai Prangphet

**Affiliations:** ^1^Department of Applied Biology, Faculty of Sciences and Liberal Arts, Rajamangala University of Technology Isan, Thailand; ^2^Department of Chemical Engineering, Faculty of Engineering, Burapha University, Thailand

## Abstract

The aril extract (0.4% g/mL) of Gac fruit in milk supplement can inhibit cancer cell lines. Moreover, the extract has no toxicity against normal cells. In a sensory test, sterilized low-fat milk supplemented with 0.4% extract did not have different sensory score compared to the control. During the sterilization process, extract was not significantly different from the control. In sterilization (121°C, 15 min), adding Gac fruit extract in low-fat milk results in antioxidant activity increase. The extract increased values for the redness and yellowness of sterilized low fat, but the lightness decreased. Also, the extract slightly decreased the alcohol stability of sterilized low-fat milk. At an accelerated rate (50°C, 28 days), there was no effect of the extract addition on protein aggregation in low-fat milk. Moreover, the TBA values indicate the ability of the extract to inhibit lipid oxidation. Finally, Gac fruit extract added to milk may possibly extend the shelf life of sterilized low-fat milk and improve its antioxidant and anticancer activity properties.

## 1. Introduction

Nowadays, people are interested in functional foods due to the fact that they are associated with a healthy and long life. Many medicinal plants are used as ingredients in these foods. *Momordica cochinchinensis* Sprenger or Gac fruit is an edible medicinal plant which is widely found in Thailand. Its major active components, which are extracted with 70% ethanol, are lycopene, carotenoids, phenolics, and flavonoids [1, 2]. Gac fruit has been previously reported to have various bioactivities such as antitumor, anticancer, and anti-inflammatory [3, 4]. According to a previous study [1], Gac fruit is a rich source of antioxidant compounds. The aril, which was extracted with 70% ethanol, had the highest 2,2-diphenyl-1-picrylhydrazyl (DPPH) scavenging activity (IC_50_ = 865 *μ*g/mL). Moreover, its seeds, which were extracted with solvents, were also reported to have anti-inflammatory, anticytotoxicity, and antitumor properties [5–8]. At the same time, the aril of Gac extracted by hot water has been reported to have antitumor and anticancer activities [9].

Many applications of plant extracts as ingredients in milk products have been previously reported. Cocoa powder reduces the sedimentation of chocolate milk [10]. Low-fat milk supplemented with tea, coffee, and cocoa extracts displays increased protein stability [11]. Aloe vera extract, oak bark, and oak leaves or coconut shell enhance the stability of low-fat milk (120°C) and concentrated milk (140°C) [12]. The rosemary and green tea extracts were tested for their effects on pasteurized low-fat milk, and it was found that milk antioxidant activity and phenolic content increased after their addition [13]. The effects of *Pueraria tuberosa* extract on pasteurization (72°C, 15 s) and sterilization (121°C, 15 min) of milk were examined, and it was found that the extract changed the chemical and physical properties of milk and works more efficiently in pasteurized milk than in sterilized milk [14]. Also, green tea extract enhanced the physical and chemical stability of milk [15].

In this study, the aril of Gac fruit was extracted by hot water and treated under 121°C for 15 min, and its antioxidant and antiproliferative activities were examined. Moreover, the effects of the extract on color, sensory attributes, and protein stability of sterilized low-fat milk were determined.

## 2. Materials and Methods

### 2.1. Chemicals and Reagents

The compounds 2,2-diphenyl-1-picrylhydrazyl (DPPH), 2,2′-azino-bis-3-ethylbenzothiazoline-6-sulfonic acid (ABTS), 6-hydroxy-2,5,7,8-tetramethylchroman-2-carboxylic acid (trolox), copper sulfate, thiobarbituric acid (TBA), 3-(4,5-dimethylthiazol-2-yl)-2,5-diphenyltetrazolium bromide (MTT), and oxalic acid were purchased from Sigma-Aldrich (St. Louis, USA). Potassium persulfate and methanol were purchased from Ajax Finechem (Taren Point, Australia). Trihydroxybenzoic acid (Gallic acids) was purchased from Fluka (Buchs, Switzerland). Folin–Ciocalteu's reagent, trichloroacetic acid (TCA), hydrochloric acid (HCl), sodium hydroxide (NaOH), isoamyl alcohol, and acetone were purchased from Carlo Erba (Val-de-Reuil, France). Hexane, methanol, sulfuric acid, acetic acid, and petroleum ether were obtained from RCI Labscan (Bangkok, Thailand). Boric acid, methyl red, and bromocresol green were purchased from Qrec Chemical Co. LTD. (Chonburi, Thailand). Sodium carbonate was purchased from Kemaus (New South Wales, Australia). Sodium Dodecyl Sulfate (SDS) was purchased from SDFCl (Sd Fine Chem Limited, Tamil Nadu, India). Dimethyl sulfoxide (DMSO) was purchased from Fisher (Pennsylvania, United States). Ammonium persulfate, tetramethyl ethylenediamine (TEMED), protein marker, and acrylamide were purchased from Bio-Rad (California, USA). Tris base was purchased from Vivantis (Selangor Darul Ehsan, Malaysia). Brilliant blue R was purchased from ACROS organics (Antwerp, Belgium). Dulbecco's Modified Eagle's Medium (DMEM) was obtained from Corning (New York, USA).

### 2.2. Gac Fruit Extract Preparation

The arils of ripe Gac fruits were dried at 50°C and were mixed with water in the ratio of 1 : 125. The solution was heated at approximately 70°C for 10 min and was filtered by using a muslin cloth and Whatman filter membrane number 1 (Maidstone, United Kingdom). The extract was dried at 50°C and kept at -20°C. The extract was then heated at 121°C for 5 min and was kept at -20°C for further study.

### 2.3. Determination of Lycopene and *β*-Carotene Content

The extract from the aril of Gac fruit (0.4% g/mL) was tested for the lycopene and *β*-carotene content by using the colorimetric method adapted from a previous report [16]. Extraction solvent (10 mL, hexane/acetone/ethanol: 50 : 25 : 25, *v*/*v*/*v*) was mixed with the sample (0.5 mL) and was stirred for 10 min. Distilled water (1.5 mL) was added to the mixture. The upper layer was collected and put in a glass tube. The solvent was evaporated, and then, hexane (500 *μ*L) was added to the sample. The sample was measured for the lycopene and *β*-carotene content by using a spectrophotometer (Thermo Scientific™ GENESYS™ UV-Vis, Vantaa, Finland) (450 nm: *β*-carotene and 470nm: lycopene). The extinction coefficient of 2560 M^−1^ cm^−1^ for *β*-carotene and 3,450 M^−1^ cm^−1^ for lycopene from previous reports was used for calculating the *β*-carotene and lycopene quantities [17, 18].

### 2.4. Determination of Total Phenolic Content

The total phenolic content was examined using a colorimetric method which was adapted from a previous study [13]. Shortly, 10% Folin-Ciocalteu reagent 375 *μ*L was mixed with 75 *μ*L of sample and 75 *μ*L of 7.5% Na_2_CO_3_ and incubated for 1 h at room temperature. After incubation, the mixture was measured at 715 nm. For milk samples supplemented with extract, the mixture was centrifuged at 3,300 rpm and then the supernatant was measured at 715 nm. The total phenolic contents (Gallic acid equivalents per gram of dry weight, mg GAE/g DW for extracts or Gallic acid equivalents per liter of samples, and mg GAE/L for milk samples) of each sample were calculated from the standard curve of Gallic acid.

### 2.5. Antiproliferative Activity

The anticancer activity of the extract was determined by MTT assay [19]. Briefly, lung cancer cells (human melanoma cell line A 375 and human lung adenocarcinoma cell line A 549) and normal cells (human skin keratinocyte cell line and normal human dermal fibroblast) were cultured in DMEM with 10% (*v*/*v*) fetal bovine serum, 100 U/mL penicillin, and 100 *μ*g/mL streptomycin. After incubation at 5% CO_2_, 37°C, for 3 days, the cells were seeded in 96-well plates with approximately 2 × 10^4^ cells per well. The extract was incubated with the cells in 5% CO_2_ at 37°C for 24 h. After incubation, the medium was replaced with the medium consisting of 0.5 mg/mL MTT reagents and was then incubated for 30 min at the condition 5% CO_2_, 37°C. After incubation, the medium of each well was replaced with 100% DMSO 150 *μ*L. The 96-well plate was further measured at 570 nm. The control was the medium without the extract. The cell viability was calculated using the following formula.

The %cell viability = ((Abs 2) × 100)/(Abs 1) where Abs 1 represents the absorbance of a negative control and Abs 2 represents the absorbance of a sample.

### 2.6. DPPH Scavenging Assay

The ability of DPPH scavenging of each sample was determined using a colorimetric method which was adapted from a previous study [13]. Shortly, 0.2 mM DPPH 550 *μ*L was mixed with 50 *μ*L of samples. The mixture was left in darkness at room temperature for 30 min. After incubation, the mixture was measured at 517 nm. For milk samples supplemented with extract, the mixture was centrifuged at 3,300 rpm before the supernatant was measured at 517 nm. The trolox equivalent of each sample was calculated from the standard curve of trolox. The % DPPH scavenging was calculated as follows. The %inhibition = ((Abs 1 − Abs 2) × 100)/(Abs 1), where Abs 1 represents the absorbance of a negative control and Abs 2 represents the absorbance of a sample.

### 2.7. ABTS Scavenging Assay

ABTS scavenging of each sample was measured using a colorimetric method which was adapted from a previous study [14]. Briefly, 7.4 mM of ABTS was mixed with 2.6 mM potassium sulfate at a ratio of 1 : 1 and incubated in darkness at room temperature for 12 h. The mixture was measured at 732 nm and then was diluted for 7.2. ABTS (1 mL) was mixed with 1 *μ*L of sample. For milk samples supplemented with extract, the mixture was centrifuged at 3,300 rpm before the supernatant was measured at 732 nm. The trolox equivalent of each sample was calculated from the standard curve of trolox. The % ABTS scavenging was calculated as follows. The %inhibition = ((Abs 1 − Abs 2) × 100)/(Abs 1) where Abs 1 represents the absorbance of a negative control and Abs 2 represents the absorbance of a sample.

### 2.8. Effect of Gac Fruit Extract on Sterilized Milk

#### 2.8.1. Sensory Test

The protocol of sensory test was determined by the Human Research Ethics Committee, Rajamankala University of Isan (Project code: HEC-01-63-217) based on the Helsinki guideline. All persons provided their informed consent prior to their inclusion in the study. The low-fat milk supplemented with the extract was prepared in 0.2% and 0.4% *v*/*v*, and the low-fat milk with no extract was tested as a control. All samples were labeled with three-digit numbers and served in random order in a private room. The 30 judges scored the samples in flavor, odor, color, and overall acceptability by using a 9-point hedonic scale (9 = like the best, 5 = neither like nor dislike, and 1 = dislike the most).

#### 2.8.2. Color

The effect of the extract on sterilized milk color was examined as previously described [20]. The color meter was calibrated with a standard white tile, as specified by the manufacturer. The samples were poured on clear Petri dishes with no bubbles and then were measured at three different positions by using Hunter *L*∗, *a*∗, and *b*∗ values by a BYK-Gardner color meter (Geretsried, Germany). Data were presented in terms of *L*∗ (lightness) ranging from 0 (black) to 100 (white), *a*∗ (redness) ranging from +60 (red) to -60 (green), and *b*∗ (yellowness) varying from +60 (yellow) to -60 (blue).

#### 2.8.3. Alcohol Stability

The protein stability was adapted from the method previously described [21]. Shortly, ethanol at various concentrations (70-100%, with a 2% increase for each level) was prepared. Ethanol (500 *μ*L) at each concentration was mixed with 500 *μ*L of the milk sample in a glass tube. After incubation for 30 min, a visible protein sediment was observed. The minimum ethanol concentration that produced a visible protein sediment was reported.

#### 2.8.4. Protein Aggregation

The low-fat milk supplemented with 0.4% extract was treated in accelerated condition (50°C, 28 days) and was examined for protein stability by using SDS-PAGE. The samples (low-fat milk with no extract (control), low-fat milk supplemented with 0.4% extract, and pasteurized milk) were mixed with 4x loading buffer and heated at 100°C for 5 min. After that, the samples were loaded in 12% SDS-PAGE and run at a condition of 120 volts for 2 h. The gel was stained with Coomassie brilliant blue. After the destain process, protein bands appeared and were analyzed for the protein stability [22].

#### 2.8.5. TBA Values

Lipid oxidation was measured by a TBA method with some modifications [23, 24]. Firstly, 0.2 g of sample was mixed with 5 mL of 20% TCA and 5 mL of distilled water were added and mixed for 10 min. The mixtures were centrifuged at 4,000 rpm for 5 min. The supernatant (0.5 mL) was mixed with 0.5 mL of 0.01 M TBA acid solution. The mixture was left in boiling water for 30 min. When the temperature of the mixture was at room temperature, the mixture was measured at 532 nm by using a spectrophotometer (Thermo Scientific™ GENESYS™ UV-Vis, Vantaa, Finland). The data were subtracted from the value of a negative control which was distilled water and were expressed as the absorbance values at 532 nm.

#### 2.8.6. HMF (Hydroxyl Methyl Furfural) Values

The Maillard reaction was determined by an HMF method with some modifications [25]. Firstly, sample (250 *μ*L) was mixed with 0.3 N oxalic acid (125 *μ*L). The mixture was left in boiling water for 30 min. After it had cooled down to room temperature, 49% TCA (125 *μ*L) was mixed into the mixture. The mixtures were centrifuged at 4,000 rpm for 5 min. The supernatant (200 *μ*L) was mixed with 125 *μ*L of 0.05 M TBA solution. The mixture was measured at 443 nm by using a spectrophotometer (Thermo Scientific™ GENESYS™ UV-Vis, Vantaa, Finland). The data were subtracted from the value of a negative control which was distilled water and were expressed as the absorbance values at 443 nm.

#### 2.8.7. Proximate Analysis

The sterilized low-fat milk supplemented with 0.4% extract was tested for its content of protein, lipid, total solid, soluble solid, and ash. The protein content of the milk was determined by the Kjeldahl method as described by the Association of Official Analytical Chemists (AOAC) [26]. Also, the samples were heated in a furnace at 550°C overnight to determine the ash content. Fat content analysis was measured by the Gerber method [27]. For total solid content, the milk samples, which were in a humidity free-aluminum can, were heated in the oven at 102 ± 2°C for 1.5 h. After that, the total solid value obtained from the constant weight was calculated for the % total solid content [26]. The soluble solid content was determined by using a refractometer (Optika HR-130, Italy).

### 2.9. Statistical Analysis

The independent sample *t*-test and one-way ANOVA analysis of variance performed in SPSS were used for statistical analysis. The treatment means were compared using a Duncan test at a significance level of *p* < 0.05.

## 3. Results and Discussion

### 3.1. Total Phenolic Content

Phenolic compounds are known as antioxidants that promote health. The chemical structures of phenolic compounds are composed of a phenol ring which can scavenge free radicals [28]. The aril of Gac fruit was extracted by 70% ethanol, and the phenolic content reported was 30.8 mg/100 g DW [1]. Moreover, the aril extracted by 80% methanol had 4.29 mg GAE/g [2].

In this study, the aril of Gac fruit extracted by hot water (70°C) at 0.2% g/mL and 0.4% g/mL was examined for the total phenolic content for two conditions, which were with a high heat (121°C, 15 min) and no high heat processing. The total phenolic content of the extract with no high heat processing was numerically higher than that of the extract with high heat processing significantly (9.23 mg Gallic/g for no high heat processing; 8.96 mg Gallic/g for high heat processing), as can be seen in Table 1. The results suggest that the temperature in the high heat process (121°C, 15 min) destroyed some phenolic content. However, the phenolic content decrease was contrasted with that of Citrus (*Citrus paradisi* Changshanhuyou) peel extract, in which it was reported that the total phenolic content increased after heat treatment [29]. However, a significant phenolic content remained which was interesting for further study in sterilized low-fat milk applications.

Also, the phenolic content of the sterilized milk supplemented with or without Gac fruit extract was determined. The results are shown in Table 1. The sterilized milk without the extract had a phenolic content (164.26 mg/L) which was consistent with that reported in a previous report [14], and this result indicated that the high heat process increased short peptides having aromatic amino acids. Comparing the total phenolic content of the sterilized milk with and without the extract (control), the phenolic content of the sterilized milk supplemented with Gac fruit (171.52 for 0.2%; 172.28 for 0.4%) was significantly higher than that of the control, as a result of the remaining heat-tolerant phenolic compounds of the extract.

### 3.2. Lycopene and *β*-Carotene Content

The aril of Gac fruits is rich in lycopene and *β*-carotene. The aril extracted by 70% ethanol showed lycopene and *β*-carotene contents of 579.3 and 621.0 *μ*g/g (DW), respectively [1]. For solvent extraction (hexane/acetone/ethanol), the extract expressed lycopene and *β*-carotene contents at approximately 7.02 and 10 mg/g, respectively [2].

In hot water extraction (70°C), the lycopene content was lower than the carotenoid content in all treatments as shown in Table 1, corresponding to previous reports [1, 2]. The lycopene and *β*-carotene contents of the extract which passed high heat processing (10.5 *μ*mol/L for *β*-carotene; 5.01 *μ*mol/L for lycopene) were lower than those of the extract with no high heat processing (47.14 mmol/L for *β*-carotene; 31.30 *μ*mol/L for lycopene) significantly indicating that the high temperature decreased lycopene and *β*-carotene levels. However, there was remaining lycopene and *β*-carotene which may contribute to anticancer activity, antioxidant activity, and shelf-life prolongation.

### 3.3. Antiproliferative Activity

Many studies have previously reported the anticancer activities of Gac fruit extract [8, 9, 30]. However, the anticancer activities of the aril extract of Gac fruit which was treated by hot water (70°C, 15 min) and passed the high heat process in this study (121°C, 15 min) have not been previously reported. The extract in various concentrations including the concentration which was mixed in low-fat milk (0.4%, 4 mg/mL) was examined for anticancer activity against human melanoma cell line A 375 and human lung adenocarcinoma cell line A 549.

**Figure 1:**
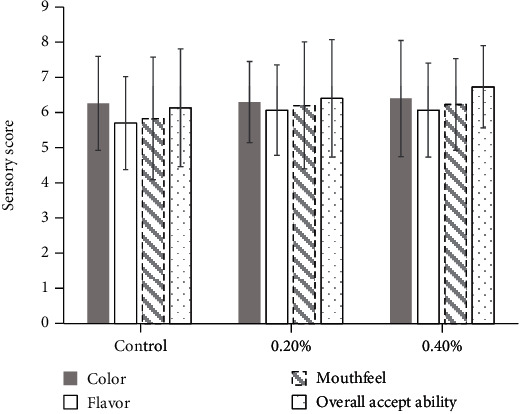
Effect of *Momordica cochinchinensis* aril extract on anticancer activities. (a) and (b) represent the toxic activities of human skin keratinocyte cell line and normal human dermal fibroblast, respectively. (c) and (d) represent the anticancer activities against human lung adenocarcinoma cell line A 549 and human melanoma cell line A 375, respectively. Data are presented as means ± SD (*n* = 5). Statistical comparisons were made between samples using one-way ANOVA (Duncan).

The results indicate that the extract at 4 mg/mL or 0.4% g/mL, which was the concentration supplemented in the milk, can inhibit the cancer cells as can be observed in Figures 1(c) and 1(d). The cell viability of human lung adenocarcinoma cell line A 549 (Figure 1(c)), which was treated with the extract at 4 mg/mL, was 70.46%, which was significantly lower than that of the control. Also, the extract at a concentration in the range of 0.25-4 mg/mL could inhibit the growth of human melanoma cell line A 375, for which the cell viability percent (64.37-89.86%) was significantly lower than that of the control. Also, the extract had no toxicity against normal cells as indicated in Figures 1(a) and 1(b). The sample treated with 4 mg/mL extract and the control had no significant difference in cell viability. Moreover, the lower extract concentrations (0.25 and 0.5 mg/mL) induced significant normal cell proliferation, as can be seen in their cell viability percent in Figure 1(b) which was significantly higher than that of the control, indicating the possibility of wound healing ability. Therefore, the low-fat milk supplemented with the extract can be an ingredient in functional foods which need high heat processing for anticancer and other purposes.

Also, the extract had no toxicity against normal cells as indicated in Figures 1(a) and 1(b). The sample treated with 4 mg/mL extract and the control had no difference in cell viability significantly. Moreover, the lower extract concentration (0.25-0.5 mg/mL) induces the normal cell proliferation significantly as can be seen in the cell viability percent of them higher than that of the control significantly indicating the possibility in wound healing. Therefore, the low-fat milk supplemented with the extract can be an ingredient in functional foods which need high heat processing for anticancer and other purposes.

### 3.4. ABTS and DPPH Scavenging Assay

The results of DPPH assay for Gac fruit extract and the milk supplemented with the extract (0.4%) are shown in Table 1. The TEAC in DPPH and ABTS assay of the extract with high heat processing (121°C, 15 min) were 2.95 *μ*M/g and 224.66 *μ*M/g, which were higher than that of the extract with hot water (70°C). These results correspond to those in some plant extracts such as Citrus (*Citrus paradisi* Changshanhuyou) and *Pleurotus sajor-cajuan,* where the increased temperature resulted in an increased antioxidant activity [29, 31]. After high heat treatment (121°C, 15 min), the extract still had antioxidant activity indicating the heat tolerance of the remaining antioxidant compounds and the lycopene conformation change. A previous study [32] has reported that lycopene can exhibit a relative isomerization change with an increase in antioxidant activity and that the cis-isomerization of lycopene has more antioxidant activity than the trans-isomerization. Moreover, heat (80°C, 240 min) results in the 13-cis isomer, and an increase in the antioxidant activity of the lycopene.

The antioxidant activities of all milk treatments were determined in ABTS and DPPH assays. The results are shown in Table 1. The results of DPPH and ABTS assay had similar trends. The adding of the extract to low-fat milk resulted in an antioxidant activity increase. The TEAC in DPPH and ABTS assay of the milk supplemented with 0.4% extract were highest, which were the highest (6.85 and 39.82 × 10^3^ M/L, respectively). The sterilized milk with no extract also had a high antioxidant activity corresponding to the findings of a previous report [14]. The TEACT in DPPH and ABTS were 2.53 and 31.41 × 10^3^ M/L, respectively, which were lower than those of the milk supplemented with 0.2% and 0.4% extract. The antioxidant activity of the sterilized milk with no extract could be explained by the heat breaking down proteins to smaller peptides and exposing aromatic amino acids, which can scavenge free radicals [33].

### 3.5. Sensory Test

**Figure 2:**
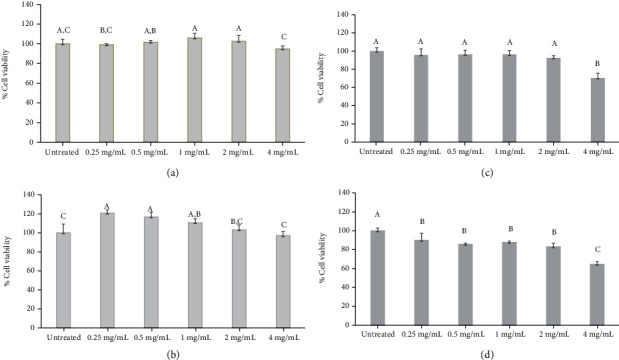
Effect of *Momordica cochinchinensis* aril extract on sensory quality of sterilized milk. Data are presented as means ± SD (*n* = 30). Statistical comparisons were made between samples using one-way ANOVA (Duncan).

The three samples (sterilized milk with no extract (control), sterilized milk with 0.2% extract, and sterilized milk with 0.4%) were evaluated by a sensory test. The results are shown in Figure 2. Even though the flavor trend of sterilized milk with 0.4% extract in all factors was higher than those of the control and the sterilized milk with 0.2% extract, all test values were not significantly different. However, the formula 0.4% extract supplement was selected for further study of chemical and physical stability, since it had the greatest antioxidant and antiproliferative activities.

### 3.6. Color

The three samples (sterilized milk with no extract (control), sterilized milk with 0.2% extract, and sterilized milk with 0.4% extract) were evaluated for the effect of extract addition on milk color. The results are presented in Table 2. The lightness index (*L*∗) of the sterilized milk supplemented with 0.4% extract decreased compared to the control (sterilized milk alone) and that supplemented with 0.2% extract. This effect corresponds to a previous report that adding *Pueraria tuberosa* in milk resulted in lightness index decrease [14]. The sterilized milk supplemented with 0.4% extract had the most intense redness index (*a*∗) among all samples. The redness index increases with a similar trend for the extract content. Lycopene, which is red and is found in the extract, may be the reason for the redness index increase. For the yellowness index (*b*∗), it has a similar pattern as determined for the redness. The higher yellowness intensity in the milk results from adding higher extract content corresponding to a previous report [14]. The carotenoid, which is yellow, may be the reason for this.

### 3.7. Alcohol Stability

Many studies have reported the effect of alcohol on milk protein stability. The sterilized low-fat milk had alcohol stability of approximately 87% as can be observed in a previous report [14]. The results of the present study are shown in Table 2. The sterilized low-fat milk had a pH ranging from 6.5 to 6.9, which was within normal range for cow milk [34]. The extract seemed to slightly reduce the ethanol stability of the sterilized low-fat milk corresponding to the previous studies [14]. However, other plant extracts such as green and black tea extracts, cocoa, and coffee powder increased the ethanol stability of skimmed milk in the pH range 6.1-7.1 [11]. On the other hand, there are no effects on ethanol stability of some plant extracts such as aloe vera extract, nondialyzable red wine, oak bark, oak leaves, or coconut shell extracts in the pH range 6.4-7.2 [12].

### 3.8. Protein Aggregation

**Figure 3:**
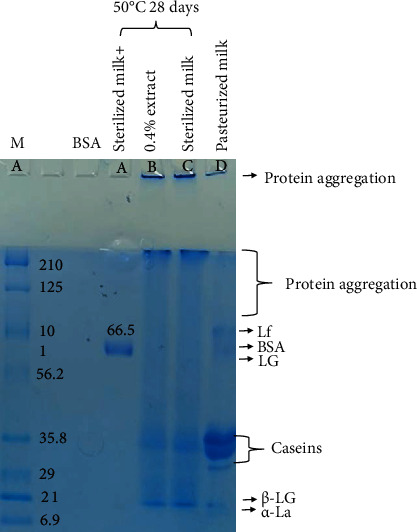
Effect of *Momordica cochinchinensis* aril extract on protein aggregation visualized by SDS-PAGE. The lanes of a marker, BSA, sterilized milk supplemented with 0.4% extract, sterilized milk alone, and pasteurized milk alone are lane A, lane B, lane C, lane D, and lane E, respectively. Lf, BSA, LG, *β*-LG, and *α*-LA stand for lactoferrin, bovine serum albumin, lactoglobulin, *β*-lactoglobulin, and *α*-lactalbumin, respectively.

The effect of the Gac extract on milk protein aggregation was analyzed by SDS-PAGE as indicated in Figure 3. Normally, the major proteins in milk are BSA (approx. 66 kDa), caseins (25-35 kDa), *β*-lactoglobulin (approx. 18 kDa), and *α*-lactalbumin (approx. 14 kDa) [35]. Protein aggregation of milk was observed clearly when it was in an accelerated condition which was 28 days of storage at 50°C [22]. After the accelerated condition, the protein band intensity of the milk control and the milk supplemented with the extract decreased and a smear in the upper part of the gel appeared, indicating protein cross-linking and aggregation. However, the smears in the upper part of the gel were not different. The result suggests that the extract supplement in milk had no major effect on the milk protein aggregation, which contrasts to the effect of green tea extract (GTE) on milk. The green tea extract resulted in protein aggregation more than that of the control. However, GTE has been reported to have the ability to extend the shelf life of milk by inhibiting proteolysis and Strecker aldehyde formation [35].

### 3.9. TBA Values

The sterilized milk supplemented with 0.4% extract was chosen for further study in the ability to inhibit the lipid oxidation under accelerated condition (40°C, 28 days). Normally, polyunsaturated fatty acids in milk generate malondialdehyde via the oxidation reaction. TBA can react with the malondialdehyde to form a colored product. The results are presented in Table 2. Comparing with the milk control, the sterilized milk supplemented with 0.4% extract had 0.0237 (Abs_532_), whereas the milk alone (control) had 0.3210 (Abs_532_). The milk supplemented with Gac fruit extract had a significantly lower TBA value. The lower TBA value indicates that the Gac fruit extract supplement in milk may induce shelf-life prolongation by inhibiting lipid oxidation. However, the TBA value determination over the long term should be performed to ensure this finding.

### 3.10. HMF Values

The Maillard reactions of all samples were determined by detecting 5-hydroxymethylfurfural (HMF), which is produced via a reduction reaction of reducing sugars [36]. The color change to brown during storage is one of the important factors associated with product, because it may affect the acceptance by consumers. In order to test the effect of the extract supplement in milk on HMF, the accelerated condition (40°C, 28 days) was performed and the milk was supplemented with 0.4% extract. The results are shown in Table 2. The absorbance values at 433 nm can indicate the HMF content of the samples [25]. In the accelerated condition, the absorbance at 433 nm of the milk supplemented with 0.4% extract was 0.547, while that of the milk alone (control) was 0.477. Also, the extract alone (0.4%) had an absorbance value of approximately 0.105. The reason why the absorbance of the milk supplemented with 0.4% extract was higher than the control milk is possibly due to sugar content of the extract. However, a long-term study may provide a more precise value.

### 3.11. Proximate Analysis

The proximate analysis of the formulation which had the best antioxidant and antiproliferative activity among the tested samples (low-fat milk added 0.4% extract) is shown in Table 3. The protein content was approximately 3.19%, corresponding to the protein content of cows' milk [37]. The lipid content was also in the lipid range of low-fat milk [38]. The low-fat milk with 0.4% extract added had a slightly higher lipid content than that of the low-fat milk alone, but it is not significant. The higher lipid content may be from the existence of lycopene and carotenoid in the extract. The total solid content of the low-fat milk with 0.4% extract was approximately 11.68%, which was slightly higher than that of the control (approximately 10.03%). The total solid content is in the range of the previous study [39]. The soluble solid of the milk supplemented with the extract was approximately 11.10% brix, which was significantly higher than that of the sterilized milk alone (10.83% brix).

## 4. Conclusions

The aril of Gac fruit extracted by hot water (70°C) and treated at 121°C for 15 min contained phenolic, lycopene, and *β*-carotene, which had antioxidant and antiproliferative activities. Also, it is possibly suitable for sterilized milk application due to lipid oxidation decrease and no major chemical and physical change. Moreover, it is suggested from this study as an ingredient in functional foods which need high heat processing.

## Figures and Tables

**Table 1:** Phenolic contents, *β*-carotene contents, lycopene contents, and antioxidant activities of Gac fruit extract and sterilized milk supplemented with Gac fruit extract.

	Gac fruit extract 0.4% alone		Sterilized milk		
	70°C	121°C	Control	0.2% extract	0.4% extract
% ABTS inhibition	2.75 ± 0.1354^A^	5.28 ± 0.7459^B^	59.22 ± 1.9004^a^	72.55 ± 2.0360^b^	75.53 ± 0.9662^b^
TEAC^∗^ (ABTS)	143.06 ± 4.3711^A^	224.66 ± 24.0743^B^	31.41 ± 0.9801^a^	38.28 ± 1.0500^b^	39.82 ± 0.4983^b^
% DPPH inhibition	0.43 ± 0.0647^A^	1.98 ± 0.1293^B^	9.50 ± 0.2580^a^	19.99 ± 1.9210^b^	29.90 ± 0.0190^c^
TEAC^∗^ (DPPH)	1.93 ± 0.0428^A^	2.95 ± 0.0856^B^	2.53 ± 0.0545^a^	4.75 ± 0.4064^b^	6.85 ± 0.4983^c^
Phenolic content^1^	9.23 ± 0.0717^A^	8.96 ± 0.0592^B^	164.26 ± 0.5696^a^	171.52 ± 0.4000^b^	172.28 ± 0.1740^c^
*β*-Carotene (*μ*mol/mL)	47.14 ± 14.3260^C^	10.5 ± 2.5981^E^	NA	NA	NA
Lycopene (*μ*mol/mL)	31.30 ± 9.0540^D^	5.01 ± 1.6696^F^	NA	NA	NA

Data are shown as means ± SD (*n* = 3). Means with different superscripts, A and B in rows are significantly different (*p* < 0.05) from each other using independent sample *t*-test. Different superscripts in rows (C, D, and E) mean the significant difference between groups (paired sample *t*-test, *p* < 0.05). Means with different superscripts (a, b, and c) in rows are significantly different (*p* < 0.05) from each other using one-way ANOVA (Duncan). Control means sterilized low-fat milk with no extract. ^∗^Represents the unit mg*ΤΕ*/g for Gac fruit extracts, and the units of 10^3^ × M/L for milk samples (ABTS) and M/L for milk samples (DPPH). ^1^Represents the unit mg Gallic/g sample for Gac fruit extracts and the unit mg Gallic/L for milk samples. NA indicates data are not available.

**Table 2:** Effect of Gac aril extract on alcohol stability, color index, TBA, and HMF values of sterilized milk.

Treatment	Ethanol stability **(**%**)**	pH	TBA^1^	HMF^2^	*L*∗	*a*∗	*b*∗
Control	87.5^A^	6.55	0.3210 ± 0.1333^D^	0.447 ± 0.0676^F^	72.76 ± 0.0451^a^	0.16 ± 0.0152^a^	5.80 ± 0.1320^a^
0.2%	85.0^B^	6.57	NA	NA	72.36 ± 0.3691^a^	1.27 ± 0.2468^b^	9.10 ± 0.4122^b^
0.4%	85.0^C^	6.61	0.0237 ± 0.0311^E^	0.54 ± 0.2194^F^	69.64 ± 0.1305^b^	1.55 ± 0.0681^c^	10.75 ± 0.1815^c^

Data are shown as means ± SD (*n* = 3). The data are expressed as the ethanol percent, which was added to the samples resulting in visual precipitation. Control was sterilized milk with no extract. Different superscripts in a column (A, B, and C) mean significant difference between groups (*p* < 0.05) by using one-way ANOVA (Duncan). Different superscripts in a column (D, E, and F) mean significant difference between groups (*p* < 0.05) by using the independent sample *t*-test. Different superscripts in a column (a, b, and c) mean significant difference between groups (*p* < 0.05) by using one-way ANOVA (Duncan). NA indicates data are not available. Superscripts of 1 and 2 indicate the absorbance values at 532 nm (TBA) and 443 nm (HMF), respectively.

**Table 3:** Proximate analysis of sterilized low-fat milk 0.4%.

Composition	Milk alone	Milk supplemented with 0.4% extract
Ash (%)	0.58^*a*^ ± 0.0220	0.60^*a*^ ± 0.0360
Total solid content (%)	10.03^*a*^ ± 0.1058	11.68^*a*^ ± 1.0347
Soluble solid (% brix)	11.10^*a*^ ± 0.0000	10.83^*b*^ ± 0.0577
Protein (%)	3.20^*a*^ ± 0.0283	3.20^*a*^ ± 0.0667
Lipid (%)	0.30^*a*^ ± 0.1000	0.53^*a*^ ± 0.1154

Data are shown as means ± SD (*n* = 3). Different superscripts in rows (a and b) mean significant difference between groups (*p* < 0.05). Statistical comparisons were made between samples using the independent sample *t*-test.

## Data Availability

The data used to support the findings of this study are available from the corresponding author upon request.

## Additional Points

*Highlights.* (i) The aril of Gac fruit was extracted by hot water (70°C, 15 min) and passed a high heat process (121°C, 15 min) but still possesses antioxidant and antiproliferative activities. (ii) The extract had the ability to extend the shelf life of sterilized low-fat milk by inhibiting lipid oxidation. (iii) The Gac extract is appropriate for the development of functional foods which need a high heat process.
